# Jumping into the 20th century before it is too late: is laboratory robotics still in its infancy?

**DOI:** 10.1155/S1463924692000142

**Published:** 1992

**Authors:** David B. Weinstein, Dennis S. France

**Affiliations:** Atherosclerosis and Vascular Biology Research Department Sandoz Research Institute E. Hanover New Jersey 07936 USA

## Abstract

Successful management of laboratory robotic automation programmes in the environment of research and drug discovery within the pharmaceutical industry may perhaps be best compared to a chef preparing the perfect hollandaise sauce. All the ingredients must be available at the same time and be of highest quality for the right price. However, if components are not added in the right quantities and in the proper order, no amount of whipping together by the product champion will create the best product. In the past, managerial scepticism surrounding useful implementation of cost-effective, high-throughput robotic systems often placed these ‘modern toys’ at low priorities for research development laboratories. Management now recognizes the unique contributions of robotics in the research environment. Although the scientific director must still play the role of product champion, new questions are being proposed and new commitments are being made to bring the potential of robotic automation to every laboratory where repetitive functions can benefit from new applications. Research laboratory directors have become both the key ingredient, as well as the rate-limiting determinant in the development of new applications. Having fulfilled the promise of robotic automation to release talented personnel, the challenge now is for the ‘end users’, the bench scientists, to be provided with opportunities to invest the time and effort required for future applications and new career functions.


Journal of Automatic Chemistry, Vol. 14, No. 2 (March–April 1992), pp. 59–63

### Jumping into the 20th century before it is too late: is laboratory robotics still in its infancy?

**David B. Weinstein and Dennis S. France**

*Atherosclerosis and Vascular Biology Research Department, Sandoz Research Institute, E. Hanover, New Jersey 07936, USA*

*Successful management of laboratory robotic automation programmes in the environment of research and drug discovery within the pharmaceutical industry may perhaps be best compared to a chef preparing the perfect hollandaise sauce. All the ingredients must be available at the same time and be of highest quality for the right price. However, if components are not added in the right quantities and in the proper order, no amount of whipping together by the product champion will create the best product. In the past, managerial scepticism surrounding useful implementation of cost-effective, high-throughput robotic systems often placed these 'modern toys' at low priorities for research development laboratories. Management now recognizes the unique contributions of robotics in the research environment. Although the scientific director must still play the role of product champion, new questions are being proposed and new commitments are being made to bring the potential of robotic automation to every laboratory where repetitive functions can benefit from new applications. Research laboratory directors have become both the key ingredient, as well as the rate-limiting determinant in the development of new applications. Having fulfilled the promise of robotic automation to release talented personnel, the challenge now is for the 'end users', the bench scientists, to be provided with opportunities to invest the time and effort required for future applications and new career functions.*

We saw him sit and try to cook.
He took a look at the book on the hook.
But a Nook can't read, so a Nook can't cook.
so . . . what good to a Nook is a hook cook book?
Every day from here to there, funny things are everywhere.

Dr Seuss [1]

Why was a biochemist involved in drug discovery in areas of cholesterol metabolism and heart disease at the podium in the early morning hours to present a keynote address to several hundred sleepy robotics specialists? What is the relevance to robotics scientists of a dedication to Dr Seuss' famous character who could not cook?

It was not the wrong time or place for the lecture – although one might have argued that biologists have been missing from International Robotics meetings and this discussion should have happened years earlier! Research biologists have been like the Nook when it comes to the use of robotics as a research tool – if you don't see or can't read about it, it may not exist. This is surprising and distressing at the same time, since many advances in current drug development efforts in the pharmaceutical industry already pay homage to automated equipment and advanced computer-assisted robotic operations. These systems are in use and provide more efficient and economical use for resources in late-stage drug formulation, stability and chemical analysis activities. The same basic elements have been available to the biologists at the front end of drug discovery, but little published literature describes any major conceptual or research advance in the use of robotics made in these laboratory settings. With the considerable investment that a pharmaceutical company makes in R&D, one should expect there to be state-of-the-art equipment and automation capabilities in every phase of drug development. If we accept the reality that biologist and research managers have been slow to adapt robotics to their research automation concepts, there must be simple reasons for this problem that can be considered before we run out of time in the twentieth century.

The average academic and pharmaceutical biologist and research manager may still consider laboratory robotics to be in the same 'magical' category as the robots of Isaac Asimov's novel or the Hollywood screen image of Woody Allen hiding in disguise as a robot butler in the movie 'Sleeper'. The average library has a wealth of information, books and magazines which preview the 'glitzy' consumer-oriented potential values of robots that play piano, make sushi and pizza, etc. [2,3]. How many university or pharmaceutical libraries have as much information devoted to robotics?

At Sandoz Research Institute, an opportunity was created for biologists to develop a drug discovery programme using robotic microplate management systems as analysis tools. This programme was accomplished with minimum amounts of funding and a staff of no more than two over a two-year period; but required an extensive learning process, as well as education of our senior research directors. Our process for adding a robotics component to a disease-oriented drug discovery process was not much different than that used by a cook making a classic hollandaise sauce or mayonnaise. Mayonnaise is a simple mixture of protein and fats and oils from eggs or other sources which when blended or whipped properly forms a *stable* emulsion which traps water and flavour ingredients [4]. In the research world, the key issues are to have available all the right ingredients (creative people + good, simple ideas + a little bit of fantasy + attainable goals) and add them together in the right quantities and the right order under the direction of the most important ingredient: the product champion. The role of the research director who is the product champion is to blend together the research and personnel components with the creative environment provided by management in order to create the successful

© Zymark Corporation 1992

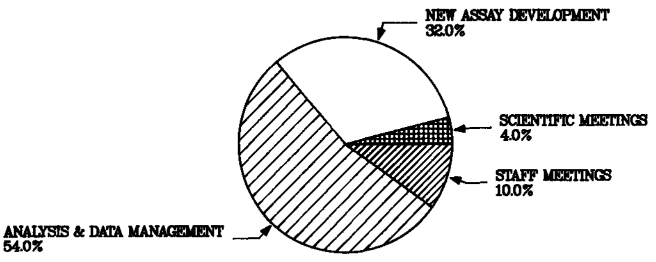

*Figure 1. Activity profile of bench scientists in 1987.*

and *stable* integration of robotics into the early stages of drug discovery. Thus, the chef making mayonnaise, and the research manager putting together a robotic research programme, require reasonably similar steps to produce the best product.

What are the components of the mayonnaise which make up the typical efforts of new drug-discovery processes within the pharmaceutical industry today? A major component is the effective team effort of both biologist and medicinal chemists in a specifically ordered sequence as a programme begins. First, the team must determine the biological site or mechanism of action which is the target of the new drug substance. Second, there must be developed a set of *in vitro* and *in vivo* test models, which can discriminate between compounds which effectively alter the biological target selected. Third, large numbers of medicinal chemists would provide rational concepts towards design of complicated chemical molecules which alter the biological regulatory mechanism selected as the drug target. This rational chemical design concept is often balanced by a well-known pharmaceutical programme called 'random-screening' in which complex biological samples from natural sources such as bacterial and fungal broths, extracts from plants, natural herbal remedies etc. are screened through assays to measure effects on cell growth, enzyme activities or complex biological behaviours. The roots of the pharmaceutical development of drugs lie in this ancient and tedious method of searching through natural products.

It has been estimated that one major new biological lead will result from 20–50 000 random screening analyses, providing that the analysis has the proper discriminating power for separating positive and false-positive or negative hits. One would like to screen a minimum of 50 000 samples a year through the appropriate assay screens in order to have a high enough rate of new lead acquisition for new programmes in drug discovery. One can readily identify the need for robotic screening and analysis in this process, which a single screening programme in one disease area requires such high throughput of repetitive sample preparation and analysis. Even with this type of biological lead in hand, it may still take several years of chemical modification and supportive biological and toxicological development before a drug may appear on the market.

A substantial commitment of effort of many skilled staff members is required for successful completion and development of drug discovery functions. An analysis of the activity profile of Sandoz's biology bench scientist for the year 1987 is shown in figure 1. New assay development for research screening and analysis accounted for approximately one third of biology staff time at the lab bench. Additionally, despite the widespread use of modern computer hardware and software it required just over 50% of the scientist's time to compile, analyse, manage, and report the data accumulated by all analysis systems. Only 15% of their time was devoted to staff and scientific meetings. However, in this pre-robotics era, our technical staff had little or no free time for new training opportunities and other creative growth programmes. Further analysis of the research programme indicated that several constant parameters, or requirements, could be deduced from the standard operating practices which defined the operations of good drug screening. First, amongst these parameters, is the requirement for a high degree of precision of measurements. Second, is the requirement that a robust assay be reproducible over long periods of time during the search for active agents. Third, is that the assay be able to discriminate between weakly-effective, as well as, strongly active compounds. Finally, the assay must have a high enough throughput to allow for a low 'hit-rate' at the early stages of development of any screen. All of these parameters were ideally suited for robotic development of new screening programmes

Figures 2 and 3 shows typical examples of the, analysis methods which have been developed in Sandoz's lipoprotein laboratory. The first (figure 2) is a serum cholesterol analysis indicating the excellent linear correlation between the final robotic assay and the manual assay from which it was developed. Figure 3 shows the consistency of performance of the assay over three years using four cholesterol reference standards. As a consequence of the introduction of two robots to perform a number of varied analytical end-points, this laboratory has experienced a continuing increase in the number of new chemical entities screened per year since 1988 (figure 4). In 1991, time spent by the staff on new assay development continued to increase (32% to 40%); however, analysis and data management time has dropped from 54% to only 10% of total effort (figure 5). Thus, 50% of total staff time is now available for new, more creative functions, managerial, training and professional activities. On the lighter side, as management has been exposed to the successful development of robotics within our research laboratory environment there had been a corresponding linear increment in the number of laboratory tours given for visiting dignitaries and consultants (figure 6).

In the past, managerial scepticism surrounding useful implementation of cost-effective, high-throughput robotic systems often placed these 'modern toys' at low priorities for research and development laboratories. Robotics, at the level of the research laboratory, has different rationales and constraints than does heavy industry robotization. For the research or clinical laboratory there is the necessity to maintain repetitive analysis of biological samples, which is most often conducted by highly skilled technical staff. Efficiency of function tends to fall and errors in analysis tend to increase in proportion to the fatigue and tedium introduced by the constant need for repetition. Coupled with the need for higher throughput in front-end biological research screening are the dynamic changes in team management concept and

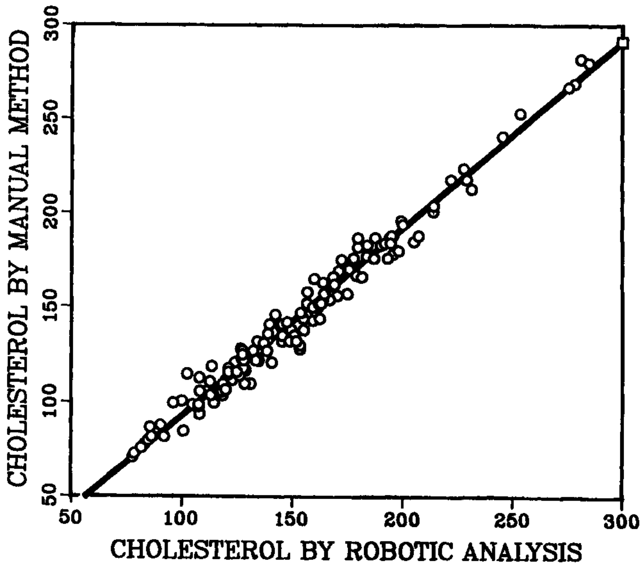

*Figure 2. Robotic analysis provides both high-quality and high-quantity biological data.*

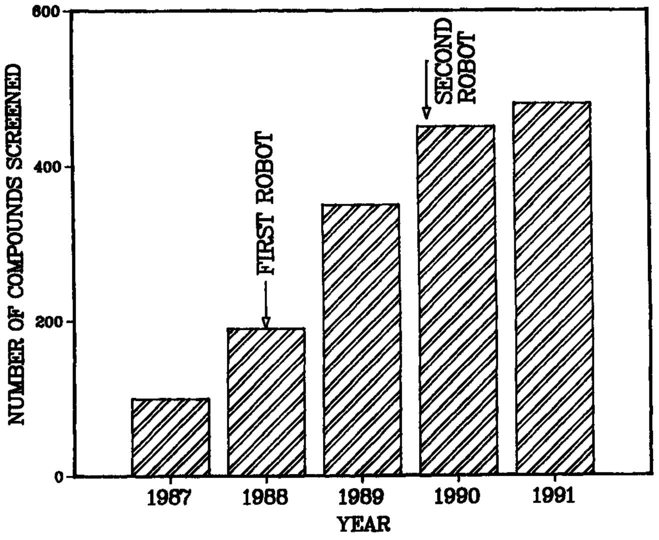

*Figure 4. Robotic automation increases number of compounds screened.*

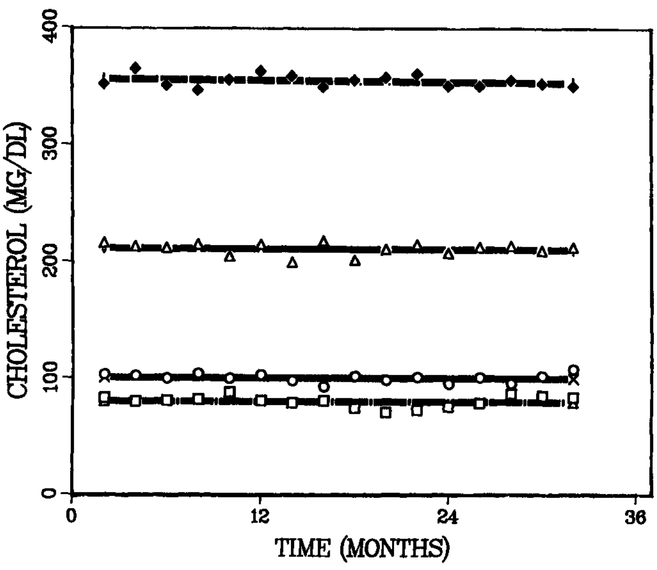

*Figure 3. Performance of quality control standards over time.*

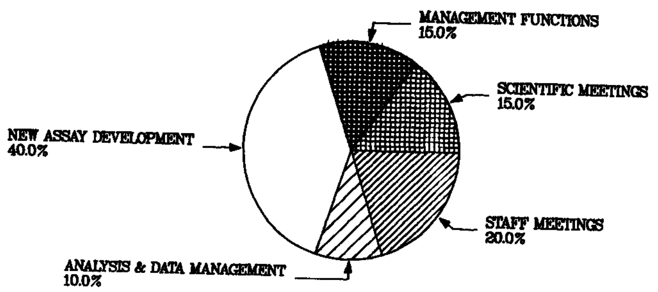

*Figure 5. Activity profile of bench scientist in 1991.*

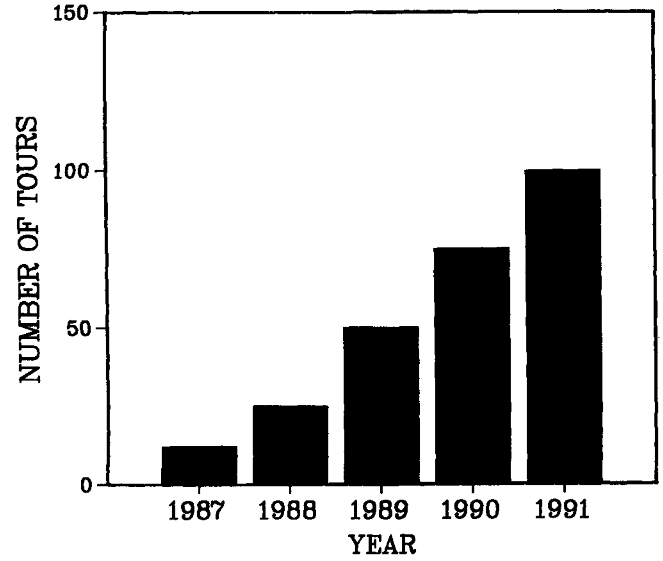

*Figure 6. Robotic systems increase number of laboratory tours.*

managerial approaches in many industrial settings. Management now recognizes the unique contribution of robotics in the research environment, which can allow highly skilled team members to pursue more creative functions than those programmes which may have committed these staff members to be tied to performance of manual repetitive tasks.

It has been suggested that Japanese industrial managers and government officials consider robots to be an essential component of industrial growth in a nation which has a severe labour shortage problem, compounded by limitations in Japan's immigration policy [2]. An opposite philosophy has existed in a number of US industrial markets in which robotic automation has been considered to be a threat to jobs that might be more appropriately or successfully accomplished by modern engineering rather than by creative staffing. Are we doing things correctly in adapting robotic and automation to the drug research and development functions of the pharmaceutical industry? Robotic technology occurs in companies of all sizes in Japan, while in the US the market appears to be left exclusively to a relatively small number of the major manufacturing industries. As the robot gap widens, the monetary investment in Japan is at a level at least six time that of US industrial investment [2], fewer industrial robots are at work in the US than the number of new robots added last year alone in Japan. Japanese robotic companies expect that one or more robots will be at work in every Japanese house in the 21st century.

While efforts to increase productivity have been the early driving force behind robotic automation, current motivation tends to develop from the scientific curiosity of exploring other planets or placing robots in environments which are hazardous to human workers for extended periods [5]. In the biological arena, it has been suggested that hospital clinical laboratories will become the site of significant growth of laboratory robotics in an effort to solve the medical technologist shortage and escalating costs of laboratory analysis [6]. A number of papers published within the past year highlight biological research applications which have been partially or completely adapted to robotic automation systems in the clinical laboratory setting. To solve staffing problems, and protect technologists from handling potentially hazardous human blood samples, the Kochi Medical School Department of Clinical Laboratory Medicine developed assays for eight hormones measured sequentially from human blood [7]. While the robot incubates samples at six different time intervals for the eight assays it performs many of the wash and reagent addition cycles. Not only does the staff avoid contact with blood, but the investigators conclude 'the robot is considered useful and indispensable for routine jobs'. Similar reports on robotics automated blood analysis routines [8] highlight the increased handling safety, optimization of smallest sample size for high throughput, flexible, serial ordering of tests, and increased quality control of data and patient identification. An interesting example of increased efficiency and throughput can be obtained in a series of comparative case studies by Ciba-Corning Diagnostics [9] reviewing five studies of hospital and reference laboratories to determine whether semi-automated immunochemical analyses could be enhanced by robotics systems. It was determined that reduction in labour costs (33–67%), and reduced turnaround time for samples occurred in all laboratories irrespective of the size of the programme with payback for the robotics systems occurring within one year in four of the five laboratories.

In the past year, a number of robotic applications have appeared in the molecular biology literature. Examples range from automation of gene expression of easily assayed enzymes, such as bacterial chloramphenicol acetyltransferase (CAT) using kinetic monitoring of the enzymatic reaction, and elimination of the chromatographic separations required in the original published method [10]. Automated detection of specific DNA sequences for both diagnostic evaluation and determination of genetic polymorphism are now becoming more numerous [11]. The applications of robotics in this field are potentially only limited by the imagination and skill of the investigators.

Robots are now being used in nearly every phase of drug development within the pharmaceutical industry. It is unfortunate that the bounds of proprietary information does not permit industrial scientists to share their experiences in robotic development, such as in application of general screening of discovery of new drug substances. An excellent example of the design strategy and benefits of automation of drug testing can be found in a new National Cancer Institute human tumour drug screen [12]. The original screening assay in mice was extremely labour intensive and was poorly predictable to human disease. After NCI switched to a screen of 60 different human cancer cells in cell culture, an automated microplate assay system was developed which achieved a greater than tenfold increase in productivity over manual methods for only twice the total cost. Robotic automation has increased the capacity to screen over 40 000 compounds per year at approximately 20% of the cost per compound in the original manual assay.

Despite the improved efficiency and increased throughput with short payback times for return on investment costs, there are still sceptics who suggest that robotic automation has limited application to clinical and research laboratories. These analyses often argue that the speed of individual operations of a robot is slow compared to that of manual methods. This is certainly true; however, on the basis of continuous operation for extended time periods of up to 24 h per day the robotic systems are undoubtedly more accurate and precise than technicians performing the same functions in a repetitive manner over a 6–8 h work shift. A key ingredient in the successful application of robotics to research laboratory problems is the integration of research staff expertise design of scanning assays which can be coupled with the engineering skills and hardware development capabilities of robotics development companies. The product of this interaction can be simpler and faster robotic operations which can be done in combination with precision batch technology – such as in the microplate technology used at the Sandoz Research Institute – with appropriate flexibility to achieve best quality and highest throughput. We must also keep in mind that it is not always practical or essential to robotize every step or procedure in a complex series of sample preparation or analysis. In the simplest case, the chef's list of ingredients for successful development should include:

(1) Creative research staff support.

(2) Co-operative engineers.

(3) Simple movements for robots.

(4) Precise movement of individual steps.

(5) Constant sample history.

(6) Flexibility of combinations of operations.

The largest asset which may be acquired through robotic automation in the environment of the research laboratory can be the creative re-investment of the talent of the people who can be freed from the tedium of continuously repetitive research operations. A goal of every research laboratory director should be to provide growth and opportunity for all staff members. To this end, one can expand the opportunities of the 'cooks' at the laboratory bench by blending together the ingredients of laboratory robotic automation with well-co-ordinated team efforts of the laboratory staff.

### Acknowledgments

The authors wish to express their thanks to Mary Murdoch, Naved Surve, Rob Quinby, Mary Russell, and Dr John Babiak and Dr James Paterniti for their contributions to research robotics development in the laboratory, and to Dr Lester Salans for providing the right management environment for development of the robotics programme at the Sandoz Research Institute.

### References

1. Dr Seuss, *One fish, two fish, red fish, blue fish.*
2. Tanzer, A. and Simon, R., *Forbes* (16 April 1990), 148–153.
3. Large pizza, hold the microchips. *Discover* (April 1991), 12.
4. Morowitz, H. J., *Hospital Practice* (October 1979), 33–34.
5. Toon, J., *Research Horizons,* **9** (Spring 1991), 3–9.
6. Hard, R., *Hospitals* (21 June, 1991), 56–57.
7. Sasaki, M. and Ogura, K., *Clinical Chemistry,* **36** (1990), 156–157.
8. Godolphin, W., Bodtkar, K., Uyeno, D. and Lek-Oon Goh, *Clinical Chemistry,* **36** (1990) 1551–1555.
9. DeMoranville, V. E. and Ellis, J. E., *Clinical Chemistry,* **36** (1990), 1588–1590.
10. Chaucchereau, A., Astinotti, D. and Bouton, M.-M., *Analytical Biochemistry,* **188** (1990), 310–316.
11. Nickerson, D. A., Kaiser, R., Lappin, S., Stewart, J., Hood, L. and Landegren, U., *Proceedings of the National Academy of Sciences USA,* **87** (1990), 8923–8927.
12. Feldman, A. Shim. S. S. and Kramer, B. M., *Discover,* **41** (1991), 251–254.

